# 

**Published:** 1908-06

**Authors:** 


PUBLISHED MONTHLY. ATLANTA SUBSCRIPTION $1.00

### JOURNAL-RECORD OF MEDICINE

LIBRARY [illegible]

Successor to { Atlanta Medical and Surgical Journal, Established 1855. and Southern Medical Record, Established 1870. } 53d Year

Vol. X. ATLANTA, GA., JUNE, 1908. No. 3

